# Efficacy and safety of standalone middle meningeal artery embolization for chronic subdural hematoma: a single-center retrospective cohort study

**DOI:** 10.1186/s12883-026-04933-2

**Published:** 2026-04-30

**Authors:** Dajiang Xie

**Affiliations:** Department of Neurosurgery, Sir Run Run Shaw Hospital, Zhejiang University School of Medicine, Hangzhou, 310016 People’s Republic of China

**Keywords:** Chronic subdural hematoma, Middle meningeal artery, Embolization, Minimally invasive surgery, Hematoma resolution, Neurological recovery, Surgical intolerance

## Abstract

**Background:**

Chronic subdural hematoma (CSDH) is a prevalent neurosurgical disorder with high clinical morbidity, particularly in the elderly, and conventional treatments are plagued by high recurrence rates and perioperative complications. This study aimed to rigorously evaluate the short- and mid-term efficacy and safety of standalone middle meningeal artery (MMA) embolization as a first-line minimally invasive treatment for CSDH patients who are intolerant to conventional surgical interventions.

**Methods:**

A retrospective cohort study was conducted on consecutively screened patients with CSDH who underwent standalone MMA embolization at a tertiary academic hospital between January 2020 and December 2025. Strict and quantified inclusion and exclusion criteria were applied to ensure cohort homogeneity. The embolization technique was standardized with Onyx 18 as the exclusive embolic agent. Primary outcomes included hematoma resolution rate, complete hematoma absorption rate, and hematoma recurrence rate at 3, 6, and 12 months of follow-up (all patients completed 12-month scheduled follow-up). Secondary outcomes comprised neurological functional recovery (modified Rankin Scale, mRS), midline shift improvement, and procedure-related complications (graded by the Clavien-Dindo classification). Hematoma volume was calculated using the ellipsoid formula, and linear regression analysis was performed to explore the correlation between initial hematoma volume and time to complete absorption (as an exploratory analysis).

**Results:**

A total of 85 patients with 99 CSDHs (14 bilateral cases) were enrolled, accounting for 18.6% of the total CSDH patients admitted to our hospital during the same period, with a mean age of 63.6 ± 9.2 years (32 patients ≥ 70 years, 37.6%). All MMA embolization procedures achieved 100% technical success with no intraprocedural conversion to open surgery. At the 12-month follow-up, the mean hematoma volume was significantly reduced from a baseline of 59.4 ± 21.8 mL to 4.1 ± 8.7 mL (*P* < 0.001, 95% CI: 51.2–59.9), and the mean midline shift was completely resolved from 2.0 ± 2.9 mm to 0 mm (*P* < 0.001, 95% CI: 1.4–2.6). The complete hematoma absorption rates were 42.4% (95% CI: 32.9–52.4), 75.8% (95% CI: 66.8–83.2), and 80.8% (95% CI: 72.2–87.4) at 3, 6, and 12 months, respectively, with a mean hematoma resolution rate of 1.84 ± 0.69 days per milliliter (95% CI: 1.69–1.99); a strong positive linear correlation was observed between initial hematoma volume and time to complete absorption in CSDHs with complete resolution (R²=0.90, *P* < 0.001), which reflects limited variability in this homogeneous subgroup rather than generalizable predictive power. Neurological function was significantly improved (*P* < 0.001, Wilcoxon signed-rank test), with 70.6% (95% CI: 59.7–79.5) of patients achieving mRS 0 and 29.4% (95% CI: 20.5–40.3) achieving mRS 1 at 12 months (no patients with mRS ≥ 2). No severe procedure-related complications (Clavien-Dindo ≥ II) were observed; only 26 patients reported mild postoperative headache, which resolved with conservative management within 3 days. Hematoma density (hypodense/mixed/hyperdense) was not associated with hematoma absorption outcomes (*P* > 0.05).

**Conclusions:**

Standalone MMA embolization with Onyx 18 is a safe and effective minimally invasive treatment for CSDH patients who are intolerant to conventional surgical interventions, yielding a 0% mid-term recurrence rate, significant hematoma resolution, and excellent neurological functional recovery in the selected cohort. This technique represents a promising alternative to conventional surgical interventions for the above-mentioned patient population. The linear correlation between initial hematoma volume and time to complete absorption provides a single-center exploratory quantitative tool for personalized patient follow-up, and its clinical value needs to be verified in multi-center cohorts.

**Trial registration:**

The study was approved by the Institutional Review Board (IRB) of Sir Run Run Shaw Hospital.

## Introduction

Chronic subdural hematoma (CSDH) is one of the most common neurosurgical disorders worldwide, with an incidence of approximately 12–18 per 100,000 person-years, and the rate rises exponentially with age to over 80 per 100,000 in adults aged > 70 years [1, 2]. CSDH is typically secondary to minor head trauma, and its pathogenesis is closely associated with the formation of a fragile neovascular network on the hematoma outer membrane, leading to persistent exudation and hematoma expansion [3, 4]. Untreated CSDH can cause progressive intracranial hypertension, neurological deficits (e.g., hemiparesis, cognitive impairment), and even mortality due to cerebral parenchymal compression, making timely and effective intervention critical [2, 5].

Conventional therapeutic strategies for CSDH include conservative management, burr-hole drainage (BHD), and craniotomy. Conservative management with anti-inflammatory and lipid-lowering drugs is only suitable for asymptomatic patients with small-volume hematomas (< 30 mL) and requires long-term close radiological follow-up, with a 15–20% risk of hematoma progression [1, 6]. BHD is the first-line surgical treatment for symptomatic CSDH who can tolerate surgery, but it is associated with a high recurrence rate of 10–30% within 6 months, often necessitating repeated surgery [2, 7]. Craniotomy, though effective for large or multiloculated CSDHs, is highly invasive and carries a high risk of perioperative complications in elderly patients with multiple comorbidities, limiting its clinical application [5, 8]. Thus, there is an unmet clinical need for a minimally invasive, safe, and effective treatment modality for CSDH patients who are intolerant to conventional surgery with a low recurrence rate.

In recent years, endovascular MMA embolization has emerged as a novel minimally invasive treatment for CSDH [4, 9]. Preclinical and clinical studies suggest that the MMA is the primary arterial blood supply to the CSDH outer membrane, and embolization may interrupt hematoma perfusion, potentially inhibit neovascular exudation, and modulate the local inflammatory microenvironment as reported in prior literature, thereby promoting hematoma resorption and reducing recurrence [4, 10]. Small-sample case series and preliminary retrospective studies have confirmed the preliminary efficacy and safety of MMA embolization, but most of these studies combined embolization with conventional surgical evacuation [7, 11]. A recent landmark study indicated that surgical evacuation combined with MMA embolization was the most beneficial treatment for CSDH patients who can tolerate conventional surgery [13], while the evidence for standalone MMA embolization as a first-line treatment for CSDH patients with surgical intolerance remains scarce. In addition, the correlation between initial hematoma volume and time to complete absorption, a key clinical parameter for patient follow-up and prognostic prediction, has not been quantitatively characterized in large standalone MMA embolization cohorts.

To address these research gaps, we conducted a single-center retrospective cohort study of 85 patients with 99 CSDHs who underwent standalone MMA embolization with Onyx 18 as the exclusive embolic agent (the largest cohort to date for this specific population). The primary objective was to evaluate the short- and mid-term efficacy and safety of this technique in CSDH patients with surgical intolerance, and the secondary objective was to establish an exploratory quantitative model for predicting the time to complete hematoma absorption based on initial volume. This study aims to provide clinical evidence for the clinical application of standalone MMA embolization as a first-line treatment for the selected CSDH population, and to supplement the existing evidence system of MMA embolization for CSDH.

## Materials and methods

### Study design and patient population

This retrospective cohort study was conducted at Sir Run Run Shaw Hospital, Zhejiang University School of Medicine, a tertiary academic neurosurgical center in China. The study was approved by the Institutional Review Board (IRB) of Sir Run Run Shaw Hospital. Owing to the retrospective nature of the study and use of fully anonymized patient data, informed consent was waived in compliance with the Declaration of Helsinki (2024 revision). No additional interventions were applied to patients, and all data were analyzed in de-identified form.

Consecutive patients with CSDH who underwent standalone MMA embolization from January 2020 to December 2025 were screened independently by two neurosurgeons; any discrepancies were resolved via multidisciplinary departmental case conference to ensure consistent eligibility adjudication. A total of 457 CSDH patients were admitted to our hospital during the same period, and 85 patients met the inclusion criteria (18.6%). The technique of MMA embolization for CSDH in our hospital was gradually developed from 2020, with 14 patients enrolled in 2020–2021 and 71 patients enrolled in 2022–2025.

Inclusion criteria (quantified): (1) Definitive diagnosis of CSDH (onset ≥ 3 weeks) confirmed by cranial CT or MRI, with symptomatic hematoma (headache, dizziness, focal neurological deficits) or progressive hematoma expansion (volume increase ≥ 10 mL within 2 weeks) on serial imaging; (2) Treatment with standalone MMA embolization as the primary therapeutic modality (no concomitant surgical evacuation); (3) Failure of conservative management (oral atorvastatin 20 mg/day + dexamethasone 0.75 mg/day for ≥ 4 weeks) in accordance with published CSDH conservative treatment protocols [6] or unsuitability for conventional surgery (BHD/craniotomy) (defined as: ≥70 years old with ≥ 2 comorbidities, severe cardiopulmonary dysfunction, coagulation dysfunction that cannot be corrected, or patient refusal of surgical treatment); (4) Complete medical records, radiological data, and 12-month follow-up information available.

Exclusion criteria (quantified): (1) Acute/subacute subdural hematoma (< 3 weeks from onset); (2) Contraindications to endovascular embolization (coagulopathy [INR > 1.5, PLT < 50 × 10⁹/L], severe allergy to iodinated contrast media, poor vascular access); (3) Concurrent intracranial vascular lesions (aneurysms, arteriovenous malformations) or other intracranial pathologies (brain tumors, intracerebral hemorrhage, hydrocephalus); (4) Multiloculated CSDHs with severe brain compression (midline shift > 10 mm); (5) Incomplete medical records or loss to follow-up before 12 months; (6) Received combined treatment of MMA embolization and surgical evacuation.

Definition of key terms: (1) Advanced age: Age ≥ 70 years; (2) Multiple comorbidities: Simultaneous combination of ≥ 2 chronic underlying diseases (hypertension, diabetes mellitus, valvulopathy, intracranial hypotension syndrome, coronary heart disease, chronic kidney disease, etc.); (3) Surgical intolerance: The definition is consistent with the inclusion criterion (3).

### Standardized MMA embolization procedure

All procedures were performed by two senior interventional neurosurgeons (with > 10 years of endovascular experience) in a hybrid operating room under general anesthesia to ensure procedural safety and patient comfort. The embolization technique was standardized for all patients, with the following steps:

1. Vascular access: Percutaneous femoral or radial artery puncture was performed using the Seldinger technique, based on the patient’s vascular anatomy (assessed by preprocedural ultrasonography).
2. Diagnostic angiography: A 5 F vertebral catheter was navigated to the external carotid artery, and digital subtraction angiography (DSA) was performed in multiple projections to visualize the MMA anatomy, confirm the MMA branches supplying the CSDH, and rule out accessory blood supply to the hematoma.
3. Microcatheter positioning: A 0.017-inch microcatheter (Echelon 10, Medtronic, USA) was super-selectively navigated to the distal segment of the MMA (proximal to the hematoma-feeding branches) over a 0.014-inch guidewire, to avoid non-target embolization of normal cranial blood vessels.
4. Embolic agent administration: Onyx 18 (Medtronic, USA) was slowly injected under real-time fluoroscopy guidance. Injection was paused every 0.2–0.5 mL for repeat DSA to assess embolization efficacy and prevent embolic agent migration. The embolization endpoint was complete occlusion of all MMA segments supplying the CSDH, with preservation of patency of adjacent normal external carotid artery branches.
5. Postprocedural management: The puncture site was compressed for hemostasis (15–20 min for femoral artery, 5–10 min for radial artery), and the patient was transferred to the neurosurgical care unit for 24 h of continuous vital sign and neurological monitoring. Symptomatic treatment (analgesics, antiemetics) was given as needed.

### Outcome assessment

All patients underwent scheduled clinical and radiological follow-up at 3, 6, and 12 months postprocedure. Imaging assessment was performed by two independent radiologists using a blind method, and neurological function assessment was performed by two independent neurosurgeons; inconsistent results were reviewed by a third senior physician.

Clinical follow-up included neurological examination and mRS assessment (0 = no deficit, 1 = minor deficit, 2 = slight disability, 3 = moderate disability, 4 = severe disability, 5 = bedridden, 6 = death); a reduction in mRS score ≥ 1 point was defined as clinically significant improvement. Radiological follow-up comprised cranial CT (non-contrast) to evaluate hematoma volume, midline shift, absorption status, and hematoma density (hypodense/mixed/hyperdense). Hematoma volume was calculated using the ellipsoid volume formula: V = (a×b×c)/2, where a = maximum longitudinal diameter, b = maximum transverse diameter, c = maximum thickness of the hematoma (all measured on cranial CT) [7].

Outcomes were defined as follows:

1. Primary outcomes.

- Hematoma resolution rate: Calculated as days required for hematoma resorption per milliliter of initial hematoma volume;
- Complete hematoma absorption rate: Proportion of CSDHs with complete disappearance on cranial CT;
- Hematoma recurrence/progression rate: Proportion of patients with hematoma reaccumulation (volume increase ≥ 10 mL) or new neurological deficits due to hematoma expansion during follow-up.

2. Secondary outcomes.

- Assessed by Modified Rankin Scale(mRS, https://captiva.neurosurgery.ufl.edu/wordpress/files/2025/03/modified_rankin_508C.pdf) (0 = no deficit, 1 = minor deficit, 2 = slight disability, 3 = moderate disability, 4 = severe disability, 5 = bedridden, 6 = death); a reduction in mRS score ≥ 1 point was defined as clinically significant improvement;
- Midline shift improvement: Absolute reduction in midline shift (measured from the septum pellucidum to the skull midline) on cranial CT;
- Procedure-related complications: Classified by the Clavien-Dindo classification [12]; only complications of grade ≥ II were defined as severe complications.

### Statistical analysis

Statistical analysis was performed using IBM SPSS Statistics 26.0 (IBM Corp., Armonk, NY, USA) and GraphPad Prism 9.0 (GraphPad Software, La Jolla, CA, USA). Normality of continuous variables was tested using the Shapiro-Wilk test; normally distributed variables were expressed as mean ± standard deviation (SD) with 95% confidence intervals (CI), and non-normally distributed variables as median (interquartile range, IQR). Categorical variables were expressed as n (%) with 95% CI. Paired t-tests were used to compare baseline and follow-up continuous variables (hematoma volume, midline shift). As an ordinal variable, mRS was analyzed using the Wilcoxon signed-rank test, reported as median score and categorical distribution. Chi-square test was used to analyze the correlation between hematoma density and complete absorption rate. Linear regression analysis was performed to explore the correlation between initial hematoma volume and time to complete absorption (exploratory analysis), with the coefficient of determination (R²) used to assess the strength of the correlation. A two-tailed P-value < 0.05 was considered statistically significant.

## Results

### Baseline demographic and clinical characteristics

A total of 85 patients with 99 CSDHs were enrolled in the study, and all patients completed 12-month scheduled follow-up (follow-up time: 365 days, range: 30–365 days). Fourteen patients (16.5%, 95% CI: 9.4–26.0) had bilateral CSDHs and underwent bilateral MMA embolization; the remaining 71 patients (83.5%, 95% CI: 74.0–90.6) had unilateral CSDHs (42 left-sided, 29 right-sided). Baseline demographic and clinical characteristics are summarized in Table 1.

**Table 1:** Baseline demographic and clinical characteristics of the study cohort (*n* = 85)

Parameter	Value (*n*, %) or Mean ± SD (Range) [95% CI]
Age (years)	63.6 ± 9.2 (45–75) [61.6–65.6]
≥ 70 years (advanced age)	32 (37.6%) [27.3–49.1]
45–69 years	53 (62.4%) [50.9–72.7]
Gender (Male/Female)	61 (71.8%) / 24 (28.2%) [61.0–80.6 / 19.4–39.0]
BMI ≥ 24 kg/m²	48 (56.5%) [45.3–67.1]
Past medical history	
Minor head trauma	79 (92.9%) [85.1–97.0]
Prior burr-hole drainage	6 (7.1%) [3.3–14.8]
Antithrombotic medication use	6 (7.1%) [3.3–14.8]
Multiple comorbidities (≥ 2)	29 (34.1%) [24.2–45.4]
Bilateral CSDH (bilateral embolization)	14 (16.5%) [9.4–26.0]
Clinical symptoms	
Headache	48 (56.5%) [45.3–67.1]
Dizziness	12 (14.1%) [7.8–23.1]
Seizure	8 (9.4%) [4.7–17.7]
Numbness	8 (9.4%) [4.7–17.7]
Gait instability	8 (9.4%) [4.7–17.7]
Asymptomatic	1 (1.2%) [0.0–6.4]
Baseline mRS score (median, IQR)	1 (1–1)
mRS 0	6 (7.1%) [3.3–14.8]
mRS 1	74 (87.1%) [78.0–92.9]
mRS 2	5 (5.9%) [2.5–13.2]
mRS 3–6	0 (0.0%)
Comorbidities	
Hypertension	43 (50.6%) [39.4–61.8]
Diabetes mellitus	18 (21.2%) [13.6–31.0]
Valvulopathy	6 (7.1%) [3.3–14.8]
Intracranial hypotension syndrome	6 (7.1%) [3.3–14.8]
No comorbidities	12 (14.1%) [7.8–23.1]

The cohort comprised 61 males (71.8%, 95% CI: 61.0–80.6) and 24 females (28.2%, 95% CI: 19.4–39.0), with a mean age of 63.6 ± 9.2 years (range: 45–75 years; 95% CI: 61.6–65.6); 32 patients (37.6%, 95% CI: 27.3–49.1) were ≥ 70 years old (advanced age), and 29 patients (34.1%, 95% CI: 24.2–45.4) had multiple comorbidities. Most patients (56.5%, 95% CI: 45.3–67.1) had a BMI ≥ 24 kg/m², and a history of minor head trauma was present in 79 patients (92.9%, 95% CI: 85.1–97.0). Only 6 patients (7.1%, 95% CI: 3.3–14.8) had a history of prior BHD and antithrombotic medication use.

The most common clinical symptom was headache (48 patients, 56.5%, 95% CI: 45.3–67.1), followed by dizziness (12 patients, 14.1%, 95% CI: 7.8–23.1), seizure (8 patients, 9.4%, 95% CI: 4.7–17.7), numbness (8 patients, 9.4%, 95% CI: 4.7–17.7), and gait instability (8 patients, 9.4%, 95% CI: 4.7–17.7); only 1 patient (1.2%, 95% CI: 0.0–6.4) was asymptomatic and diagnosed incidentally on cranial imaging. At baseline, the median mRS score was 1 (IQR: 1–1); mRS score was 0 in 6 patients (7.1%, 95% CI: 3.3–14.8), 1 in 74 patients (87.1%, 95% CI: 78.0–92.9), and 2 in 5 patients (5.9%, 95% CI: 2.5–13.2); no patients had severe neurological deficits (mRS 3–6). Comorbidities were common in the cohort, with hypertension being the most prevalent (43 patients, 50.6%, 95% CI: 39.4–61.8), followed by diabetes mellitus (18 patients, 21.2%, 95% CI: 13.6–31.0), valvulopathy (6 patients, 7.1%, 95% CI: 3.3–14.8), and intracranial hypotension syndrome (6 patients, 7.1%, 95% CI: 3.3–14.8).

### Baseline radiological characteristics of CSDHs

Radiological characteristics of the 99 CSDHs are presented in Table 2. The mean maximum thickness of the hematoma was 11.1 mm (95% CI: 10.2–12.0), with a mean initial volume of 59.4 ± 21.8 mL (range: 22.5–128.7 mL; 95% CI: 55.1–63.7). Delamination was observed in 9 CSDHs (9.1%, 95% CI: 4.7–16.6), and 36 patients (42.4%, 95% CI: 32.0–53.4) had a significant midline shift (≥ 1 mm, range:1–8.5 mm). Hematoma density: 67 hypodense (67.7%, 95% CI: 57.8–76.2), 28 mixed (28.3%, 95% CI: 19.8–38.5), 4 hyperdense (4.0%, 95% CI: 1.5–10.1). All CSDHs were located in the cerebral convexity, involving the frontal (*n* = 68), temporal (*n* = 52), parietal (*n* = 45), and occipital (*n* = 18) lobes (multiple lobe involvement was common). The mean baseline midline shift was 2.0 ± 2.9 mm (range: 0–8.5 mm; 95% CI: 1.4–2.6). Chi-square test showed no significant correlation between hematoma density and complete absorption rate at 12 months (*P* > 0.05).

**Table 2:** Baseline radiological characteristics of chronic subdural hematomas (*n* = 99 CSDHs)

Parameter	Value (*n*, %) or Mean ± SD [95% CI]
Laterality (patients)	
Bilateral	14 (16.5%) [9.4–26.0]
Left-sided	42 (49.4%) [38.4–60.5]
Right-sided	29 (34.1%) [24.2–45.4]
Imaging features (CSDHs)	
Mean maximum thickness (mm)	11.1 [10.2–12.0]
Mean initial volume (mL)	59.4 ± 21.8 [55.1–63.7]
Mean midline shift (mm)	2.0 ± 2.9 [1.4–2.6]
Patients with significant midline shift (≥ 1 mm)	36 (42.4%) [32.0–53.4]
Delamination	9 (9.1%) [4.7–16.6]
Hematoma density	
Hypodense	67 (67.7%) [57.8–76.2]
Mixed	28 (28.3%) [19.8–38.5]
Hyperdense	4 (4.0%) [1.5–10.1]
Lobar involvement (CSDHs)	
Frontal	68 (68.7%) [58.7–77.1]
Temporal	52 (52.5%) [42.2–62.6]
Parietal	45 (45.5%) [35.5–55.9]
Occipital	18 (18.2%) [11.7–27.1]

Note: Multiple lobe involvement was common in CSDHs; Hematoma density was not associated with 12-month complete absorption rate (*P* > 0.05)

### Perioperative and technical outcomes

All 85 MMA embolization procedures (14 bilateral, 71 unilateral) achieved 100% technical success (95% CI: 95.8–100), with complete occlusion of all hematoma-feeding MMA segments confirmed by postembolization DSA. No intraprocedural complications (e.g., vasospasm, vessel perforation, non-target embolization) were observed, and no patients required conversion to open surgery (BHD/craniotomy) during or immediately after the procedure. The mean duration of the embolization procedure was 65.2 ± 18.5 min (range: 35–120 min; 95% CI: 61.2–69.2), and the mean hospital stay was 7.8 ± 2.4 days (range: 5–14 days; 95% CI: 7.3–8.3) in Table 3.

**Table 3:** Perioperative, hematoma resolution, neurological and complication outcomes (*n* = 85 patients, 99 CSDHs)

Parameter	Value (*n*, %) or Mean ± SD (Range) [95% CI]
Perioperative outcomes	
Technical success rate	85 (100.0%) [95.8–100]
Mean procedure duration (min)	65.2 ± 18.5 (35–120) [61.2–69.2]
Mean hospital stay (days)	7.8 ± 2.4 (5–14) [7.3–8.3]
Follow-up time (days)	365 (30–365)
Hematoma resolution outcomes	
Baseline mean hematoma volume (mL)	59.4 ± 21.8 [55.1–63.7]
12-month mean hematoma volume (mL)	4.1 ± 8.7* [2.3–5.9]
Baseline mean midline shift (mm)	2.0 ± 2.9 [1.4–2.6]
12-month mean midline shift (mm)	0.0*
Mean hematoma resolution rate (days/mL)	1.84 ± 0.69 (0.79–3.74) [1.69–1.99]
Complete hematoma absorption rate (CSDHs)	
3 months	42 (42.4%) [32.9–52.4]
6 months	75 (75.8%) [66.8–83.2]
12 months	80 (80.8%) [72.2–87.4]
Hematoma recurrence/progression rate	0 (0.0%)
Neurological functional recovery	
Baseline median mRS score (IQR)	1 (1–1)
12-month median mRS score (IQR)	0 (0–1)
12-month mRS score (patients)	
0	60 (70.6%) [59.7–79.5]
1	25 (29.4%) [20.5–40.3]
2–6	0 (0.0%)
Procedure-related complications	
Severe complications (Clavien-Dindo ≥ II)	0 (0.0%)
Mild postoperative headache (Clavien-Dindo I)	26 (30.6%) [21.4–41.3]
Other complications (fever, seizure, puncture site hematoma)	0 (0.0%)

*P < 0.001 vs. baseline (paired t-test for continuous variables; Wilcoxon signed-rank test for mRS)

### Hematoma resolution and radiological outcomes

**Fig. 1:**
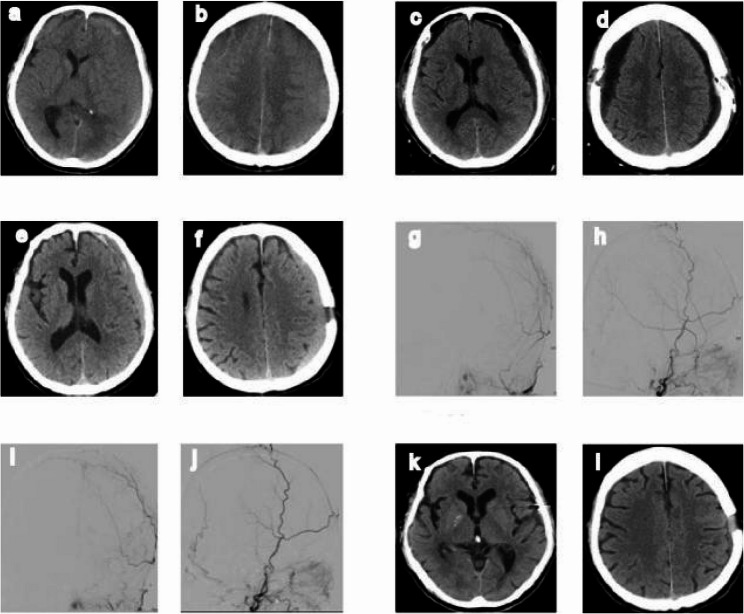
Middle meningeal artery embolization for recurrent chronic subdural hematoma after drilling and drainage. **a**-**f** and **k**-**l** are computed tomography figures, **g**-**j** are digital subtraction angiography figures. **a** and **b** indicate bilateral chronic subdural hematoma, **c** and **d** are pictures reviewed after drilling and drainage, **e** and **f** indicate recurrence of chronic subdural hematoma on the left side. The left middle meningeal artery can be seen in **g** and **h**, and **i** and **j** suggest that the middle meningeal artery is embolized. **k** and **l** are the left chronic subdural hematoma that has been absorbed

**Fig. 2:**
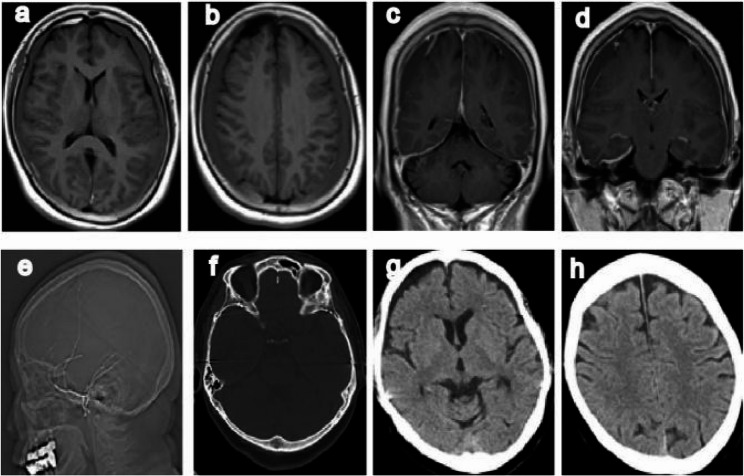
Middle meningeal artery embolization for chronic subdural hematoma caused by intracranial hypotension syndrome. **a**-**d** are magnetic resonance images, suggesting chronic subdural hematoma and dural enhancement, and **e**-**h** are computed tomography images, suggesting bilateral occlusion of the anterior and posterior branches of the middle meningeal artery and complete hematoma absorption

Hematoma resolution and radiological outcomes at 3, 6, and 12 months of follow-up are summarized in Table 3. Even cases of chronic hematomas associated with recurrence after drilling and low intracranial pressure headaches can be cured ((Figs. 1 and 2). At the 12-month follow-up, the mean hematoma volume was significantly reduced from a baseline of 59.4 ± 21.8 mL to 4.1 ± 8.7 mL (*P* < 0.001, 95% CI: 51.2–59.9), and the mean midline shift was completely resolved from 2.0 ± 2.9 mm to 0 mm (*P* < 0.001, 95% CI: 1.4–2.6). All 36 patients with a significant baseline midline shift achieved complete resolution of midline shift by the 6-month follow-up.

**Fig. 3:**
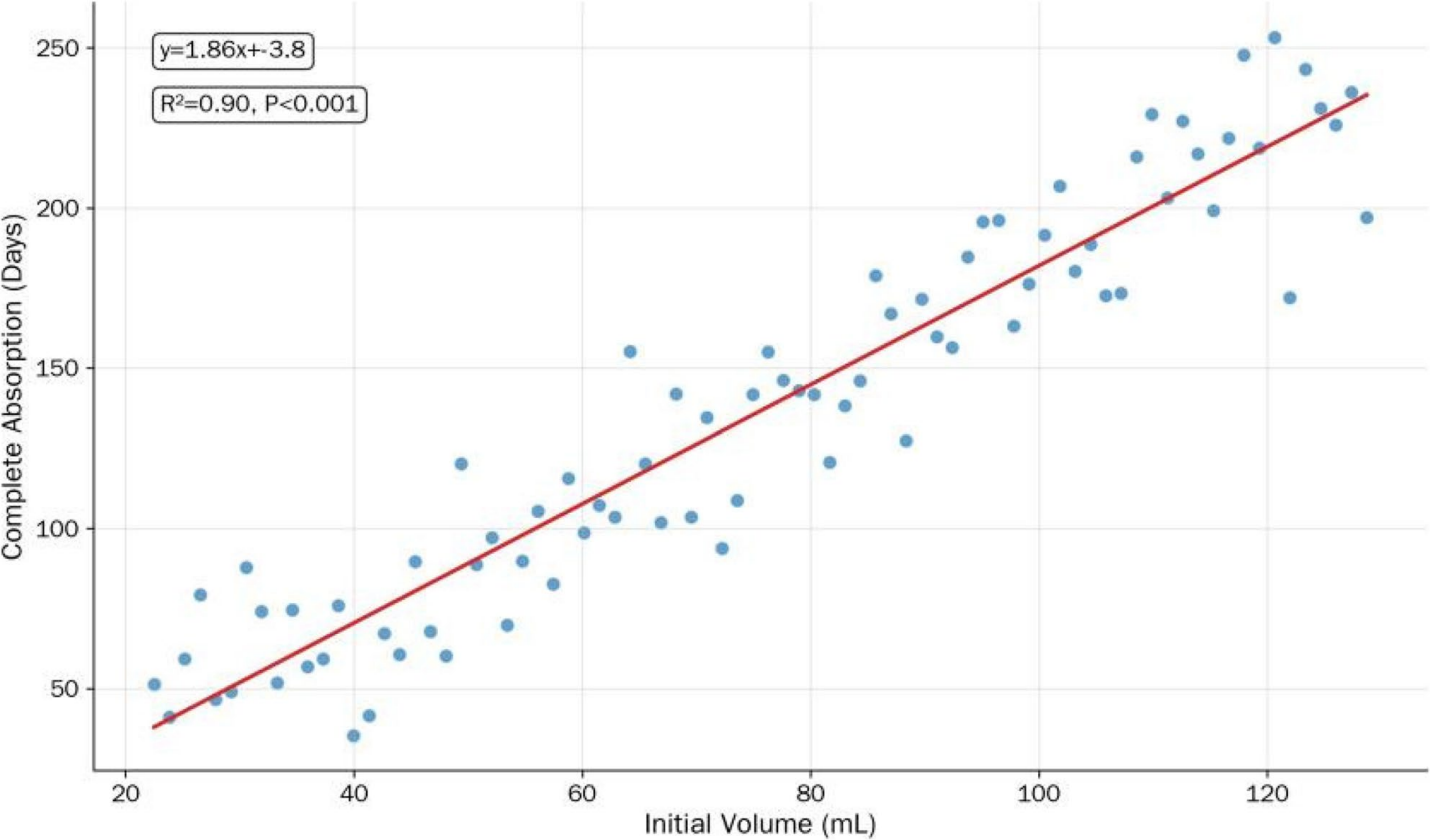
Linear Regression Analysis of Initial Hematoma Volume and Time to Complete Absorption in CSDH after Standalone MMA Embolization

The complete hematoma absorption rate showed a time-dependent increase: 42 of 99 CSDHs (42.4%, 95% CI: 32.9–52.4) at 3 months, 75 of 99 (75.8%, 95% CI: 66.8–83.2) at 6 months, and 80 of 99 (80.8%, 95% CI: 72.2–87.4) at 12 months. The remaining 19 CSDHs (19.2%, 95% CI: 12.6–27.8) exhibited significant partial absorption (volume reduction > 90%), with no hematoma expansion or clinical deterioration observed. Linear regression analysis (exploratory) revealed a strong positive linear correlation between initial hematoma volume and time to complete absorption in the 80 CSDHs with complete resolution (R²=0.90, *P* < 0.001; Fig. 3), with a mean hematoma resolution rate of 1.84 ± 0.69 days per milliliter (range: 0.79–3.74 days/mL; 95% CI: 1.69–1.99). This high R² value reflects the narrow baseline variability and homogeneity of this selected subgroup, rather than robust generalizable predictive performance for unselected CSDH populations. No hematoma recurrence or progression was recorded in any patient during the 12-month follow-up period.

### Neurological functional recovery

Neurological functional recovery assessed by mRS at baseline and 12 months of follow-up is shown in Table 3. Wilcoxon signed-rank test showed a significant improvement in median mRS score from baseline (1, IQR 1–1) to 12 months (0, IQR 0–1) (*P* < 0.001). At baseline, 7.1% (95% CI: 3.3–14.8) of patients had mRS 0, 87.1% (95% CI: 78.0–92.9) had mRS 1, and 5.9% (95% CI: 2.5–13.2) had mRS 2. At the 12-month follow-up, 60 patients (70.6%, 95% CI: 59.7–79.5) achieved mRS 0 (no neurological deficit), and 25 patients (29.4%, 95% CI: 20.5–40.3) achieved mRS 1 (minor deficit). No patients had an mRS score ≥ 2 at the 12-month follow-up. All patients with baseline seizure (*n* = 8) and gait instability (*n* = 8) achieved complete resolution of these symptoms by the 6-month follow-up, with no recurrence during subsequent follow-up.

### Procedure-related complications

No severe procedure-related complications (Clavien-Dindo ≥ II) were observed in any patient during the perioperative period or 12-month follow-up (Table 3). The only adverse event was mild postoperative headache, reported in 26 patients (30.6%, 95% CI: 21.4–41.3) within 24 h of the procedure. This headache was mild (visual analog scale score 2–4/10, https://methods.sagepub.com/ImageData/DownloadImageData? imageUrl=https://stpltrsrcscmnprdwus001.blob.core.windows.net/rsrcs/srm/images/sage-encyclopedia-of-educational-research-measurement-evaluation/10.4135_9781506326139-fig185.jpg) and resolved completely with oral non-steroidal anti-inflammatory drugs (ibuprofen 200 mg tid) within 3 days; it was classified as a Clavien-Dindo grade I complication and not considered a clinically significant adverse event. No other complications (e.g., fever, seizure, contrast-induced nephropathy, puncture site hematoma, thromboembolism) were recorded in any patient.

## Discussion

This single-center retrospective cohort study is one of the largest series to date evaluating the efficacy and safety of standalone MMA embolization with Onyx 18 as the exclusive embolic agent for CSDH patients who are intolerant to conventional surgery. The key findings of this study are: (1) 100% technical success rate with no intraprocedural complications or conversion to open surgery; (2) significant hematoma resolution with a time-dependent complete absorption rate (80.8% at 12 months, 95% CI: 72.2–87.4) and a 0% mid-term recurrence/progression rate; (3) statistically significant neurological functional recovery with all patients achieving mRS ≤ 1 at 12 months; (4) an extremely low rate of mild perioperative complications with no severe adverse events; (5) hematoma density is not associated with absorption outcomes; (6) a strong positive linear correlation between initial hematoma volume and time to complete absorption is observed in an exploratory analysis, with the high R² driven by subgroup homogeneity rather than broad predictive utility. These results confirm that standalone MMA embolization is a safe and effective minimally invasive treatment for the selected CSDH population, and provide an exploratory quantitative tool for personalized patient follow-up.

### Efficacy of standalone MMA embolization for CSDH with surgical intolerance

The 0% mid-term recurrence/progression rate in this study is a notable finding, which is superior to the 10–30% recurrence rate of conventional BHD reported in large-scale studies [2, 7, 8]. As proposed in prior mechanistic studies, this finding may be related to interruption of the blood supply to the neovascular network on the hematoma outer membrane, which is thought to be the root cause of CSDH formation and recurrence [4, 10]. Conventional BHD only evacuates the existing hematoma but does not address the persistent exudation from the neovascular network, leading to high recurrence rates. In contrast, MMA embolization completely interrupts the blood supply to the neovascular network, which is hypothesized to inhibit further exudation and rebleeding—the primary drivers of hematoma recurrence [3, 10]. It is important to note that the 0% recurrence rate in this study is based on the selected cohort of CSDH patients with surgical intolerance, and cannot be directly extrapolated to all CSDH patients. A recent study indicated that surgical evacuation combined with MMA embolization is the optimal treatment for CSDH patients who can tolerate surgery [13], and the results of this study are complementary to the study, filling the evidence gap for standalone MMA embolization in CSDH patients with surgical intolerance [14].

The hematoma resolution outcomes in this study are also promising. The mean hematoma volume was reduced by > 90% at 12 months, with a complete absorption rate of 80.8% (95% CI: 72.2–87.4), which is comparable to that of conventional BHD (80–85%) [1, 7], and the mean midline shift was completely resolved in all patients. The exploratory linear regression analysis revealed a strong positive correlation between initial hematoma volume and time to complete absorption (R²=0.90), with a mean resolution rate of 1.84 days/mL (95% CI: 1.69–1.99). This high explanatory power is limited to this homogeneous subgroup and should not be overinterpreted as a validated predictive tool for unselected patients. This quantitative index is a novel contribution of this study to the clinical literature, as it allows clinicians to develop personalized follow-up plans for the selected cohort (e.g., more frequent CT scans for large-volume hematomas). However, this model is only a single-center exploratory analysis and has not been validated in external cohorts, so its clinical application needs to be further verified.

### Neurological functional recovery and safety

All patients in this study achieved statistically significant neurological functional recovery, with 70.6% (95% CI: 59.7–79.5) achieving mRS 0 and 29.4% (95% CI: 20.5–40.3) achieving mRS 1 at 12 months. As suggested by clinical observations, this excellent recovery rate may be due to: (1) the rapid resolution of cerebral parenchymal compression due to hematoma resorption and midline shift correction; (2) the minimal invasiveness of the MMA embolization procedure, which avoids the cerebral parenchymal manipulation and intraoperative trauma associated with conventional open surgery [3, 9]. Notably, all patients with baseline seizure and gait instability achieved complete resolution of these symptoms by the 6-month follow-up, further confirming the neurological benefits of this technique for the selected cohort. Although the baseline neurological function of the study population was mild (mRS 0–1 accounted for 94.1%), the significant improvement in the proportion of mRS 0 (from 7.1% to 70.6%) confirmed the clinical value of MMA embolization in improving the quality of life of patients.

The safety profile of standalone MMA embolization in this study is exceptional, with no severe procedure-related complications and only mild postoperative headache in 30.6% (95% CI: 21.4–41.3) of patients (resolved with conservative management). This high safety is due to the standardized super-selective embolization technique used in this study: the microcatheter was positioned in the distal MMA (proximal to the hematoma-feeding branches), and Onyx 18 was injected slowly with repeated DSA assessments to prevent non-target embolization. Onyx 18, a non-adhesive liquid embolic agent, is an ideal choice for MMA embolization because it allows for precise control of embolization efficacy and minimizes the risk of embolic agent migration [7, 15], which is a key advantage over particulate embolic agents (e.g., polyvinyl alcohol particles) [10, 16]. The absence of severe complications in this study confirms that standalone MMA embolization is a safe treatment option for elderly CSDH patients with multiple comorbidities.

### Comparison with conventional CSDH treatments

This study modified the comparison of standalone MMA embolization with conventional treatments to a literature-based indirect clinical comparison (Table 4), and clearly emphasizes that this study has no control group, and the comparison results are only for clinical reference. Standalone MMA embolization offers distinct advantages for CSDH patients with surgical intolerance: compared with conservative management, it accelerates hematoma resorption and neurological recovery, and significantly reduces the risk of hematoma progression [5, 6]; compared with BHD, it has a lower recurrence rate, avoids the need for repeated surgery, and has a similar hospital stay [2, 7]; compared with craniotomy, it is far less invasive, has a lower risk of perioperative complications, and is suitable for patients who cannot tolerate open surgery [5, 8]. For CSDH patients who can tolerate conventional surgery, surgical evacuation combined with MMA embolization is the optimal treatment option [13], and standalone MMA embolization is not recommended for this population.

**Table 4:** Literature-Based Indirect Comparison of Standalone MMA Embolization and Conventional CSDH Treatments

Characteristic	Standalone MMA Embolization (This study)	Conservative Management[1,6]	Burr-Hole Drainage[2,7]	Craniotomy[5,8]
Applicable population	CSDH with surgical intolerance	Asymptomatic, small-volume CSDH	Symptomatic CSDH with surgical tolerance	Large/multiloculated CSDH with surgical tolerance
Invasiveness	Minimally invasive (endovascular)	Non-invasive	Minimally invasive (surgical)	Highly invasive
Recurrence rate	0% (12 months) [0–4.2]	15–20% (progression)	10–30% (6 months)	5–10%
Perioperative complication rate	Extremely low (no severe complications)	None	Low (5–8%)	High (15–20%)
Neurological recovery	Significant improvement (all mRS ≤ 1 at 12 months)	Slow	Moderate	Moderate (delayed by trauma)
Mean hospital stay (days)	7.8 ± 2.4 [7.3–8.3]	Long (outpatient follow-up)	6–9	10–14

Note: This is an indirect clinical comparison based on existing literature; no head-to-head randomized controlled study was conducted in this research

### Mechanistic insights into MMA embolization for CSDH

 According to published preclinical and clinical data, the therapeutic efficacy of MMA embolization for CSDH is thought to be mediated by three core synergistic pathways [4, 10, 17]: (1) Interruption of hematoma perfusion: Embolization occludes the MMA branches supplying the CSDH outer membrane, interrupting hematoma perfusion and inhibiting further hematoma expansion; (2) Modulation of the local inflammatory microenvironment: Preclinical studies suggest that MMA embolization reduces inflammatory cell infiltration and pro-inflammatory mediator release, inhibiting neovascular formation and enhancing macrophage phagocytosis; (3) Elimination of rebleeding risk: Embolization removes the blood supply to the fragile neovascular network, potentially reducing microbleeding risk and preventing hematoma reaccumulation. These mechanisms are supported by prior investigations [3, 9] but were not directly tested in this retrospective observational study.

### Predictors of hematoma resolution

This study identified initial hematoma volume as the primary exploratory predictor of time to complete absorption, with a strong linear correlation between these two variables (R²=0.90). As noted above, this high R² is driven by the homogeneity of the study subgroup and does not indicate generalizable predictive performance. Hematoma density (hypodense/mixed/hyperdense) was not found to be associated with hematoma absorption outcomes (*P* > 0.05). Other potential predictors (e.g., age, gender, comorbidities, hematoma laterality, delamination) were not found to have a significant impact on hematoma resolution in this cohort, which may be due to the strict inclusion and exclusion criteria used to ensure cohort homogeneity (e.g., exclusion of patients with severe midline shift > 10 mm or multiloculated CSDHs). Previous studies have reported that MMA trunk diameter, midline shift degree, and antithrombotic drug use are potential risk factors for embolization failure [10, 18]; however, no embolization failure was observed in this study, likely due to the standardized super-selective embolization technique and exclusive use of Onyx 18. Larger multi-center studies are needed to further explore the predictors of treatment response in a more heterogeneous cohort of CSDH patients.

### Study limitations

This study has several inherent limitations that should be acknowledged when interpreting the results, consistent with retrospective single-center studies: (1) Retrospective design: This design may introduce selection bias and information bias, despite the use of strict inclusion and exclusion criteria and independent screening/assessment to minimize these biases; (2) Single-center cohort: The study was conducted at a single tertiary academic hospital with experienced interventional neurosurgeons, which may limit the generalizability of the results to smaller hospitals or centers with less experienced operators; (3) Selected study population: The study excluded patients with severe midline shift (> 10 mm), multiloculated CSDHs, or > 75 years old, and the conclusions are only applicable to CSDH patients with surgical intolerance, and cannot be extrapolated to all CSDH patients; (4) Exploratory predictive model: The linear regression model of initial hematoma volume and time to complete absorption is a single-center exploratory analysis, with the high R² reflecting subgroup homogeneity rather than robust validation, and cannot be used as a routine clinical prediction tool for the time being; (5) Lack of a control group: The study did not include a control group of patients treated with conventional BHD, precluding a head-to-head comparison of efficacy and safety between MMA embolization and the first-line surgical treatment; (6) Short follow-up time: The maximum follow-up time was 12 months, and the long-term efficacy and safety of standalone MMA embolization (e.g., hematoma recurrence beyond 12 months, late complications) cannot be evaluated.

### Future research directions

Based on the findings of this study and the current state of the art, future research on MMA embolization for CSDH should focus on the following key areas, with particular attention to filling the gaps in the current study:

1. Prospective multi-center randomized controlled trials (RCTs): Large-sample RCTs comparing standalone MMA embolization with conservative management for CSDH patients with surgical intolerance are urgently needed to confirm the superiority of MMA embolization in terms of recurrence rate, complication rate, and neurological recovery. In addition, RCTs comparing surgical evacuation combined with MMA embolization with standalone surgical treatment for conventional CSDH patients should be further expanded to verify the conclusions of the NEJM study in more populations.
2. Long-term follow-up studies: Follow-up studies of ≥ 3 years are needed to evaluate the long-term recurrence rate, neurological functional recovery, and late complications of standalone MMA embolization, and to confirm its sustained efficacy and safety in the long term.
3. Evaluation in high-risk patient populations: Future studies should evaluate the efficacy and safety of MMA embolization in high-risk CSDH patients excluded in this study, including those with severe midline shift (> 10 mm), multiloculated CSDHs, recurrent CSDH, bilateral large-volume CSDHs, and ultra-elderly patients (> 75 years) with multiple comorbidities, to expand the applicable population of MMA embolization.
4. Validation and optimization of the predictive model: Based on the exploratory linear regression model established in this study, multi-center external validation should be carried out, and more influencing factors (e.g., MMA trunk diameter, inflammatory factor levels) should be included to optimize the model, so as to develop a clinically applicable prognostic model for predicting the time to complete hematoma absorption.
5. Comparison of embolic agents and techniques: Head-to-head studies comparing the efficacy and safety of different embolic agents (e.g., Onyx 18, polyvinyl alcohol particles, n-butyl cyanoacrylate) and different embolization techniques (e.g., proximal vs. distal MMA embolization) are needed to optimize the embolization procedure and improve treatment outcomes for different CSDH subgroups.

## Conclusion

This single-center retrospective cohort study demonstrates that standalone middle meningeal artery embolization with Onyx 18 as the exclusive embolic agent is a highly safe and effective minimally invasive treatment for chronic subdural hematoma patients who are intolerant to conventional surgical interventions. This technique achieves a 100% technical success rate (95% CI: 95.8–100), significant hematoma resolution (80.8% complete absorption at 12 months, 95% CI: 72.2–87.4), a 0% mid-term recurrence/progression rate, and statistically significant neurological functional recovery (all patients with mRS ≤ 1 at 12 months), with no severe procedure-related complications observed in the selected cohort.

Standalone MMA embolization represents a promising alternative to conventional surgical interventions (burr-hole drainage, craniotomy) for CSDH patients with surgical intolerance, and is particularly suitable for elderly patients with multiple comorbidities who cannot tolerate open surgery. The strong linear correlation between initial hematoma volume and time to complete absorption is observed in a homogeneous subgroup, with the high R² value limited to this selected population, providing a single-center exploratory quantitative tool for personalized patient follow-up and prognostic prediction. Its clinical application value needs to be verified in multi-center cohorts.

The results of this study complement the existing clinical evidence system of MMA embolization for CSDH, and are consistent with the conclusions of the latest landmark study that surgical combined with interventional therapy is the optimal option for conventional CSDH patients. Future prospective multi-center RCTs with long-term follow-up are needed to confirm the long-term efficacy and safety of standalone MMA embolization, validate the predictive model, and evaluate its efficacy in high-risk CSDH populations, so as to further establish its clinical application status as a first-line treatment for CSDH patients with surgical intolerance.

## Data Availability

The datasets used and/or analyzed during the current study are available from the corresponding author on reasonable request, with the approval of the IRB of Sir Run Run Shaw Hospital.

## Abbreviations

CSDH: Chronic Subdural Hematoma
MMA: Middle Meningeal Artery
DSA: Digital Subtraction Angiography
mRS: Modified Rankin Scale
CT: Computed Tomography
MRI: Magnetic Resonance Imaging
BMI: Body Mass Index
RCTs: Randomized Controlled Trials
